# Supplementary material for: Molecular and Photosynthetic Performance in the Yellow Leaf Mutant of Torreya grandis According to Transcriptome Sequencing, Chlorophyll a Fluorescence, and Modulated 820 nm Reflection
Source: Cells. 2022 Jan 27;11(3):431. doi: 10.3390/cells11030431 (PMC8834079; doi:10.3390/cells11030431)
Supplement: Supplementary file 1 [file cells-11-00431-s001.zip › Figures and Table s1-s4.pdf]

Supplementary Material

Supplementary Figures and Tables

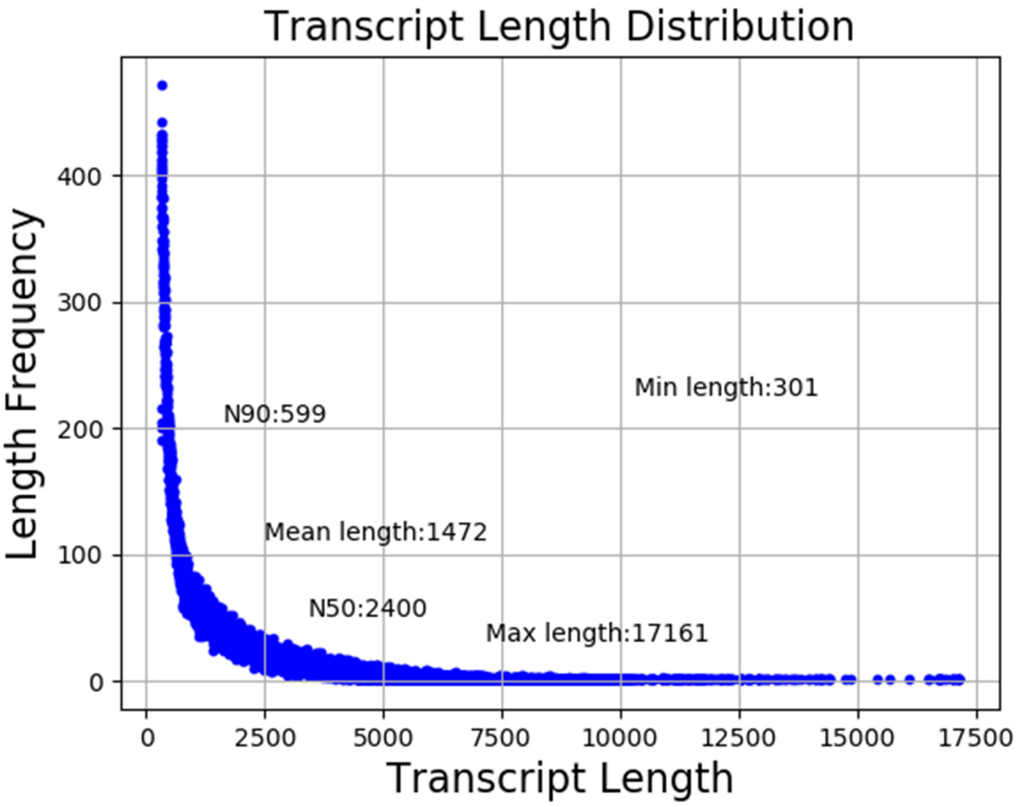

Figure S1 Assembly results in the leaves of mutant type and wild type of *Torreya grandis*.

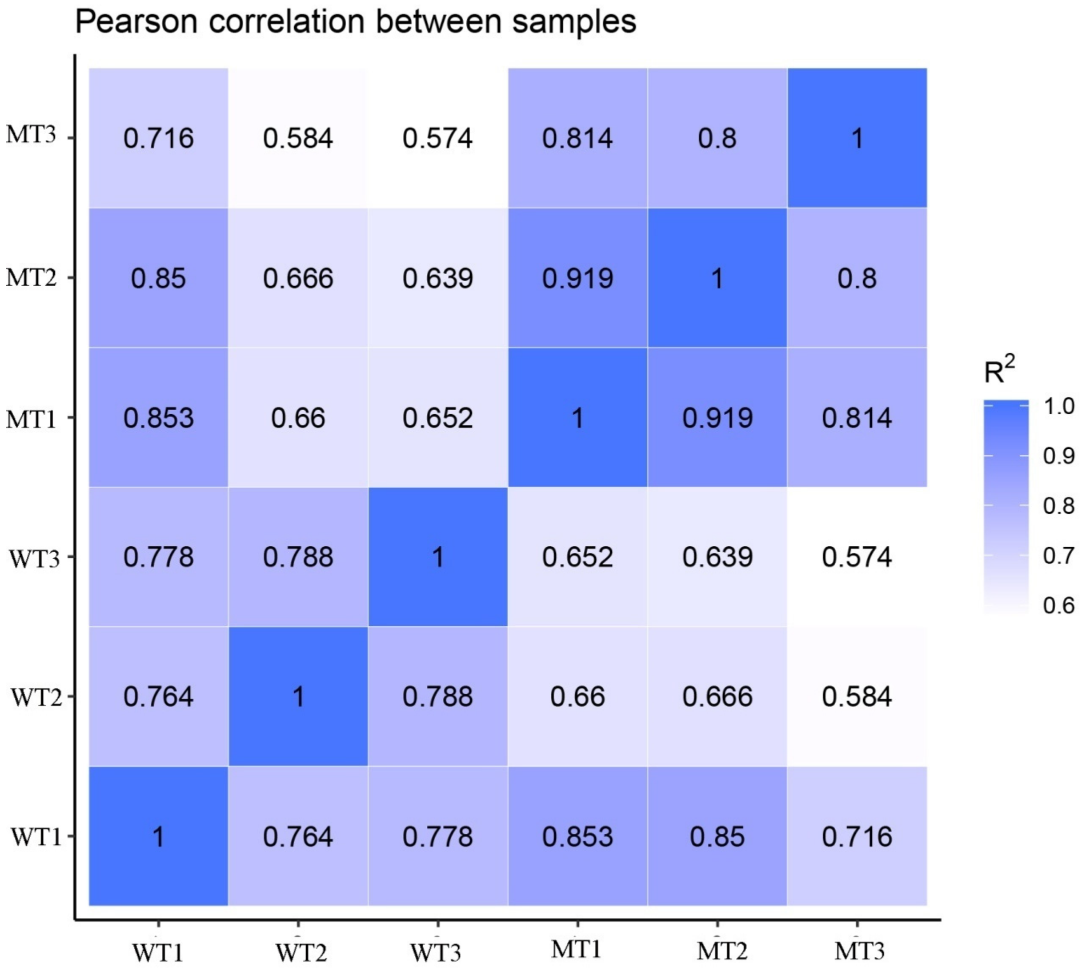

Figure S2 Pearson's correlation coefficients between two biological replicates in the six samples.

(WT1, WT2, and WT3 are the three biological repeats of the wild type of *Torreya grandis*; MT1, MT2, and MT3 are the three biological repeats of the mutant type of *T.grandis*)

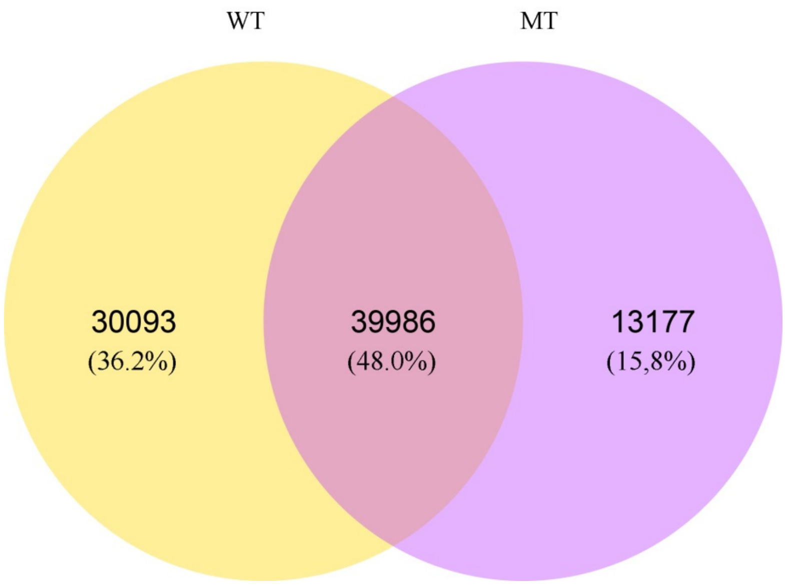

Figure S3 Venn diagram of the number of DEGs (Padj<0.05) in the leaves of mutant type and wild type of *Torreya grandis.* (WT means wild type; MT means yellow leaf mutant type)

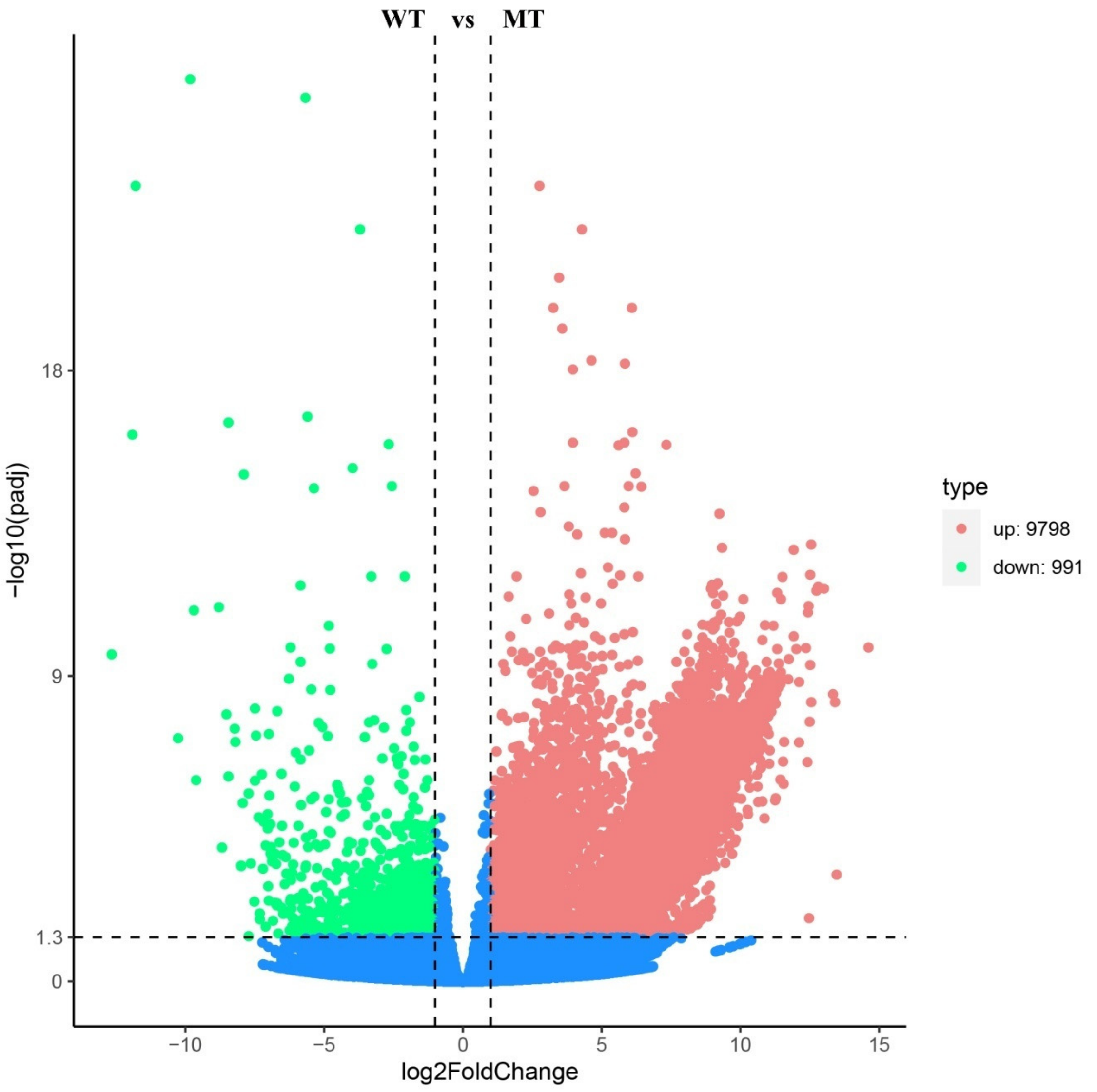

Figure S4 Volcano map of the DEGs in leaves of mutant type and wild type of *Torreya grandis*.((WT means wild type; MT means yellow leaf mutant type)

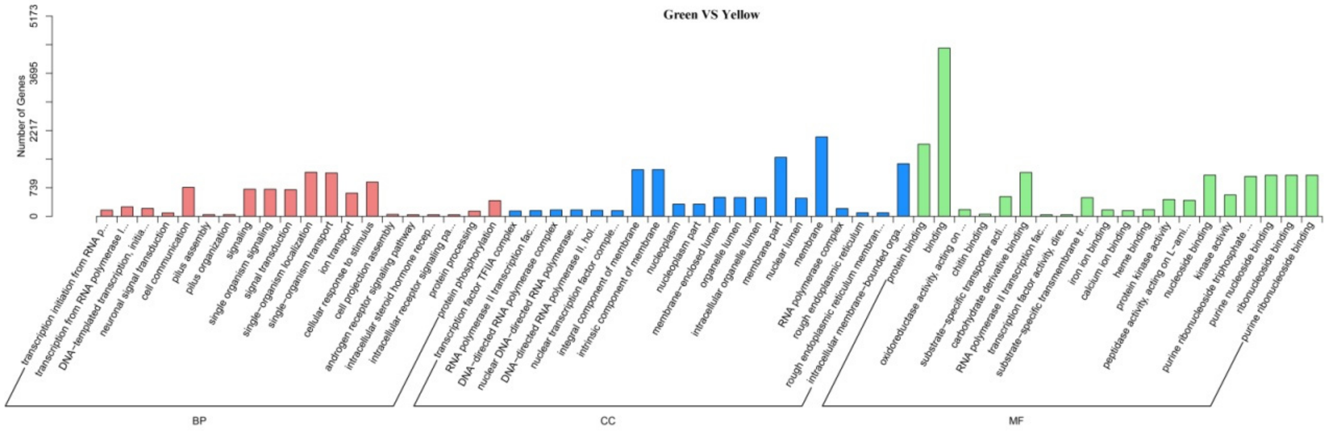

Figure S5 GO annotation classification frequencies in leaves of the leaves of mutant type and wild type of *Torreya grandis*.

(BP, biological processes; MF, molecular function; CC, cellular component)

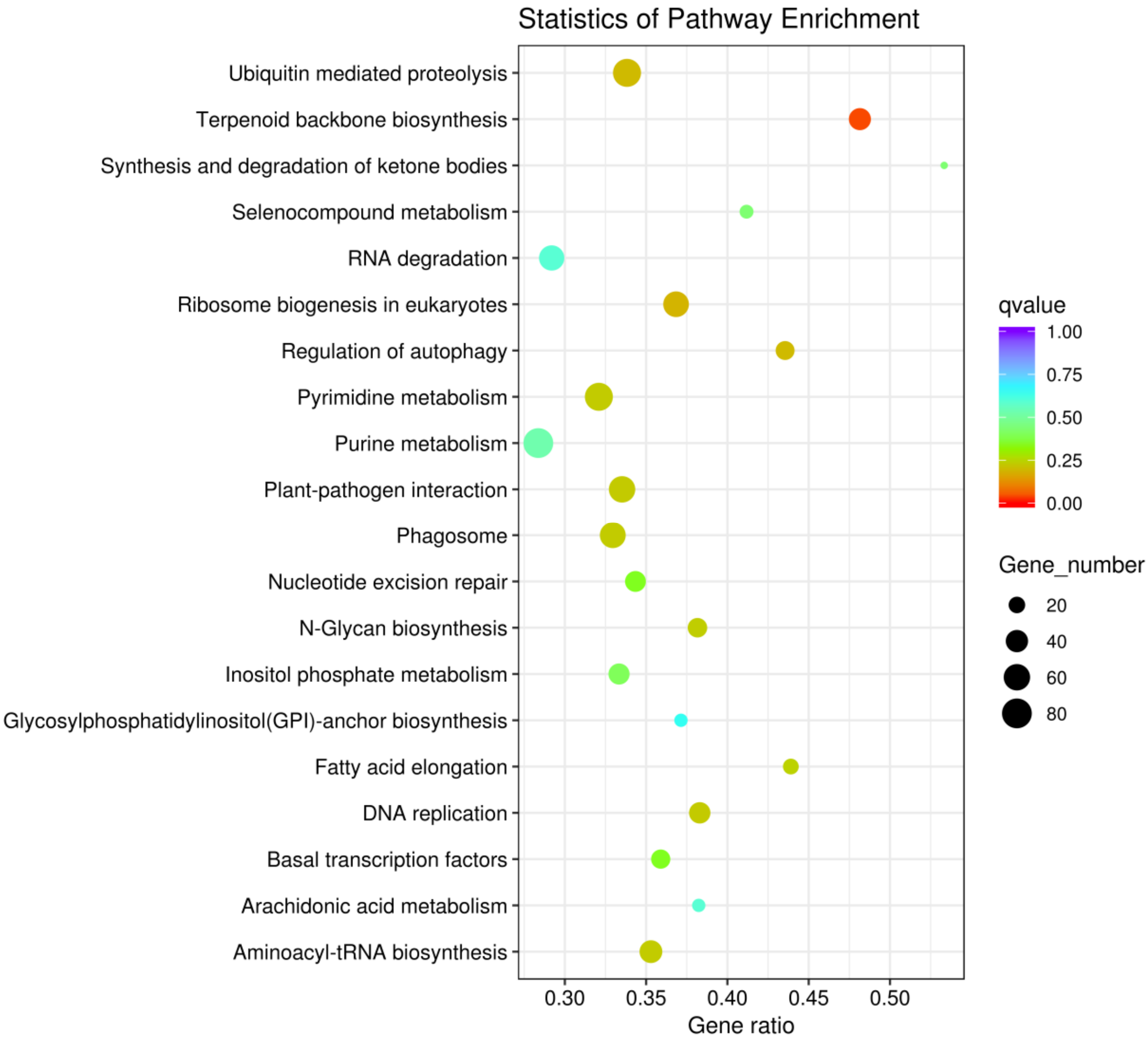

Figure S6 KO category enrichment of up/down regulated DEGs in leaves of the leaves of mutant type and wild type of *Torreya grandis*. (The number of genes in each category is equal to the dot size. The dot color represents the q-value.)

Table S1: Upper and lower primer sequences of genes evaluated using qPCR experiments.

Gene	Unigen ID	Upper primers	Lower primes
Cyclophilin (CYP)	Cluster-41647.20988	GGAGTGATGCCAGTGAGAAA	ATTTGGTGGAGATGGGAGATG
chlL	Cluster-41647.30461	GGAAGGTATGGCTCCCAAATTA	CATCGCAGCTAACACAGTATCT
hemE	Cluster-41647.18465	CCCAGCCTGCCATTAATTCT	TCAACTGTCCAATCCAGACTAAC
EARS	Cluster-41647.32963	CTGGGATTAGACTGGGATGAAG	AGCAGCATCTCCGCATATT
VDE	Cluster-41647.29473	CTGGTCAATAGAGGAGGTTCAG	CAGCATTGTTCCCGAGTTAGA
petH	Cluster-41647.15332	CAGGAGACAAGGTCCAGATTAC	CCAGTTCCAGTGGCTATCATTA
psbA	Cluster-41647.24000	GTATTCGGCGGCTCTCTATTT	CCTGCATTAGCGGACTCATT

Table S2: Summary of sequence analysis of 6 libraries

Sample	Raw Reads	Clean Reads	Clean Bases	Error(%)	Q20(%)	Q30(%)	GC Content(%)
WT1	152501536	146351032	10.98G	0.02	98.16	94.06	43.42
WT2	152161892	143308456	10.75G	0.02	98.26	94.32	43.44
WT3	150322284	142257584	10.67G	0.02	98.38	94.57	43.12
MT1	154792396	145770368	10.93G	0.02	98.31	94.52	43.27
MT2	149870256	140573088	10.54G	0.02	98.28	94.37	43.25
MT3	152335668	143390084	10.75G	0.02	98.4	94.62	43.53

(WT means wild type; MT means yellow leaf mutant type)

Table S3: Number of total clean reads of the 6 samples mapped to reference sequences

Sample	Total reads	Total mapped
WT1	73175516	59205218(80.91%)
WT2	71654228	58128048(81.12%)
WT3	71128792	58230858(81.87%)
MT1	72885184	59632590(81.82%)
MT2	70286544	57556466(81.89%)
MT3	71695042	57895684(80.75%)

(WT means wild type; MT means yellow leaf mutant type)

Table S4: Success rate statistics of transcript annotation using seven databases.

Databases	Number of Unigenes	Percentage (%)
Annotated in NR	37250	41.4
Annotated in NT	17620	19.58
Annotated in KO	89955	100
Annotated in SwissProt	30947	34.4
Annotated in PFAM	33717	37.48
Annotated in GO	33713	37.47
Annotated in KOG	13004	14.45
Annotated in all Databases	6093	6.77
Annotated in at least one Database	89955	100

Note: Nr - NCBI non-redundant protein sequences; Nt - NCBI non-redundant nucleotide sequences; KO - KEGG Ortholog database; SwissPort - A manually annotated and reviewed protein sequence database; Pfam –Protein family; GO – Gene Ontology; KOG – Clusters of Orthologous Groups of proteins.

Table S5: The expression values of transcript data in 6 samples.
